# Analysing the dynamics of the bacterial community in pozol, a Mexican fermented corn dough

**DOI:** 10.1099/mic.0.001355

**Published:** 2023-07-06

**Authors:** Rafael López-Sánchez, Diana Hernández-Oaxaca, Alejandra Escobar-Zepeda, Blanca Ramos Cerrillo, Agustin López-Munguía, Lorenzo Segovia

**Affiliations:** ^1^​ Departamento de Ingeniería Celular y Biocatálisis, Instituto de Biotecnología, Universidad Nacional Autónoma de México (UNAM), Cuernavaca, Morelos, CP 62210, Mexico; ^2^​ Microbial Informatics Team, European Bioinformatics Institute (EMBL-EBI), Cambridge, UK

**Keywords:** pozol, maize, corn, metagenomics, CAZy, bioinformatics

## Abstract

Pozol is a traditional prehispanic Mexican beverage made from fermented nixtamal dough; it is still part of everyday life in many communities due to its nutritional properties. It is the product of spontaneous fermentation and has a complex microbiota composed primarily of lactic acid bacteria (LAB). Although this is a beverage that has been used for centuries, the microbial processes that participate in this fermented beverage are not well understood. We fermented corn dough to produce pozol and sampled it at four key times to follow the community and metabolic changes (0, 9 24 and 48 h) by shotgun metagenomic sequencing to determine structural changes in the bacterial community, as well as metabolic genes used for substrate fermentation, nutritional properties and product safety. We found a core of 25 abundant genera throughout the 4 key fermentation times, with the genus *Streptococcus* being the most prevalent throughout fermentation. We also performed an analysis focused on metagenomic assembled genomes (MAGs) to identify species from the most abundant genera. Genes involving starch, plant cell wall (PCW), fructan and sucrose degradation were found throughout fermentation and in MAGs, indicating the metabolic potential of the pozol microbiota to degrade these carbohydrates. Complete metabolic modules responsible for amino acid and vitamin biosynthesis increased considerably during fermentation, and were also found to be abundant in MAG, highlighting the bacterial contribution to the well-known nutritional properties attributed to pozol. Further, clusters of genes containing CAZymes (CGCs) and essential amino acids and vitamins were found in the reconstructed MAGs for abundant species in pozol. The results of this study contribute to our understanding of the metabolic role of micro-organisms in the transformation of corn to produce this traditional beverage and their contribution to the nutritional impact that pozol has had for centuries in the traditional cuisine of southeast Mexico.

## Data Summary

Raw data from shotgun sequencing are available under BioProject number PRJNA648868. The code used for all bioinformatic analyses and complete results for each can be found at: https://github.com/RafaelLopez-Sanchez/pozol_shotgun.git

**Impact Statement**

Pozol is a traditional Mexican beverage that still plays an important role in the diet of many southern Mexican communities. This beverage serves as a significant source of carbohydrates and is particularly beneficial given that, historically, it has been a common choice for workers enduring long strenuous work days. Not only does it help alleviate hunger, it also keeps people well hydrated thanks to its refreshing properties. Prepared from nixtamalized corn, pozol delivers the same amount of protein and fibre that one would obtain by consuming a comparable portion of tortillas. When consumed in its fermented state, pozol is rich in beneficial micro-organisms. A detailed description of its bacterial community and some characteristics of its metabolic properties, particularly its potential for carbohydrate transformation, may contribute not only to understanding this complex corn fermentation process, but also to looking at this richness as a niche of biodiversity. The presence of complete metabolic modules responsible for the biosynthesis of essential amino acids and vitamins explains the well-established place of pozol in traditional Mexican cuisine.

## Introduction

For thousands of years, humans have optimized conditions to promote certain types of microbial communities that are key to the conservation, safety, texture, taste and smell of fermented foods [1]. Fermentation is an important contributor to the human diet because, among other reasons, it only requires cheap technology and transforms food to a more stable form [2]. Although experimentation with complex fermented foods is relatively easy, the difficulty of isolating individual species to characterize their role in an ecosystem is a significant barrier [1]. Such roles are relevant since microbial communities metabolize sugars and other carbon sources in the food substrate, leading to a wide variety of metabolites, for example organic acids such as lactic and acetic acids and alcohols such as ethanol, and the release of certain amino acids, among other things [3]. Thus, it is not surprising that culture-independent studies have helped to change the way we study microbial ecology, considering the population as a consortium, and that retrieving information from microbial genomes directly from metagenomes, through bioinformatics tools, has led to monitoring of strain processes during fermentation [4, 5].

Corn, as with other cereals and their fermented products, is a source of important nutrients for humankind around the world. Its fermentation mainly involves lactic acid bacteria (LAB) and yeast, which improve its nutritional value and prolong its shelf life [6].

Pozol is a spontaneous, acid, nonalcoholic fermented beverage derived from nixtamal maize dough produced in the southeast of Mexico since prehispanic times; it represents an important part of the daily caloric diet and is one of the traditional components of the Mayan gastronomic culture [7]. Nixtamalization of maize consists of cooking white or yellow corn in a 1 % lime solution (w/v) (calcium hydroxide). After cooking corn, the next step is to wash it with water and grind it into a dough called nixtamal; nixtamalization reduces the relatively low concentration of mono- and disaccharides in maize, and starch becomes the main and more readily available carbohydrate. Then the nixtamal dough is moulded and wrapped in banana leaves, where the fermentation process occurs at room temperature for a few hours to several days. Once the dough has fermented, it is suspended in water [8, 9].

**Fig. 1.**
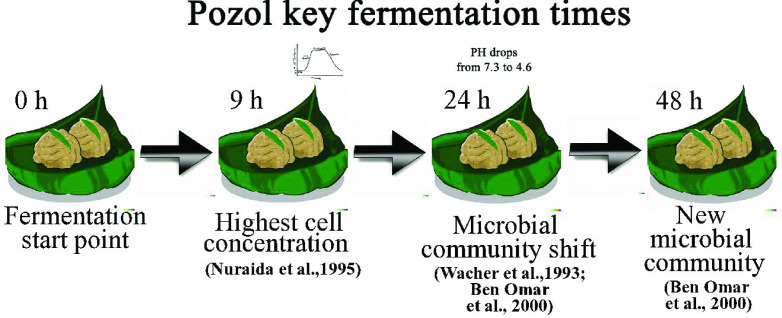
Scheme of the four key times in pozol fermentation.

The pozol microbiota is also formed mainly by LAB, fungi, and yeast, and the grinding process in local or commercial mills has been shown to inoculate the dough [10]. Previous studies have found that LAB accounted for most of the total active microbiota [11], while the genera *Enterococcus *, *Lactococcus *, *Leuconostoc *, *Streptococcus* and *Weissella* have been reported in both culture-dependent and -independent studies [11–15]. Some studies have shown that *Streptococcus* species control the entire fermentation process, in particular *Streptococcus infantarius* ssp. species. S. *infantarius* 25 124, which has already been isolated from pozol, is reported to be the main amylolytic LAB in this fermentation [14]. Other microbial analyses based on 16S bacterial rRNA genes show a significant change in community structure after 24 h of culture [13] .The pH of corn dough during pozol fermentation drops from an initial value of 7.3 to 4.6 after 30 h of incubation, suggesting that the fermentation shift occurs due to the replacement of heterofermentative LAB by homofermentative LAB [10, 11] (Fig. 1). This change in the microbial population could also be a consequence of substrate availability. Because the rather low concentration of mono- and disaccharides in maize is further reduced during nixtamalization, starch becomes the main and more readily available carbohydrate for lactic acid fermentation. However, the presence of nonamylolytic LAB indicates that starch is not the only carbon source available to sustain the microbiota present in pozol [14].

One hypothesis is that the transient accumulation of maltooligosaccharides during fermentation could presumably serve as an alternative energy source for nonamylolytic species in pozol fermentation. This would explain the observed diversity and the dominance of nonamylolytic LAB at the end of fermentation [14]. On the other hand, hemicellulose (arabinoxylan in maize) becomes available after the alkaline treatment involved in the nixtamalization and can also be metabolized by LAB. In this regard, enzyme activities, such as xylanase, ß-glucosidase, ß-arabinosidase and acetyl xylan esterase, have been reported in *S. infantarius* 25 124 [16], and secondary activities such as ß-xylosidase were demonstrated in strains of *Weissella confusa* also isolated from pozol [17].

Fermented foods have been associated with health benefits due to the presence of important nutrients such as vitamins, essential amino acids, active peptides and organic acids, as well as exopolysaccharides with soluble fibre and prebiotic properties, synthesized mainly by LAB [18, 19]. For example, a significant increase in the amount of threonine, lysine, isoleucine, tryptophan, arginine and riboflavin was reported in fermented pozol dough [20].

Despite the large number of studies dealing with pozol microbiology, the entire microbial community and its genic potential responsible for this traditional spontaneous fermentation have not been analysed using a genomic approach. In this work, we present a metagenomic study using whole-metagenome shotgun sequencing carried out at four key known crucial times during pozol fermentation. Through this approach, we describe the dynamics of the bacterial community involved over the course of fermentation and the genes related to the enzymes responsible for the degradation of dough carbohydrate (CAZymes), as well as those involved in the biosynthesis of essential amino acids and vitamins.

## Methods

### Pozol fermentation and sampling

Pozol samples were prepared as described by Rizo *et al*. [9]. Freshly ground nixtamal dough samples were obtained from two producers at the Pino Suárez market in Villa Hermosa Tabasco, Mexico. The samples were mixed and shaped into 300 g balls, wrapped in banana leaves and incubated in a humid chamber at 37 °C. Sampling was carried out at four key fermentation times 0, 9, 24 and 48 h, and was handled aseptically. The samples were placed in sealed plastic bags and stored at −70 °C . The samples were split in three batches and used in metaproteomic [9], 16S metagenomic analyses [21] and metagenomic analyses (this study).

The genomic DNA of the four fermentation samples was extracted and purified from 5 g of pozol corn dough using the PowerSoil DNA Isolation kit (catalogue no. 12888–50), PowerMax Soil DNA Isolation kit (catalogue no. 12988–10 y) and UltraClean Microbial DNA Isolation kit (catalogue no. 12224–250) following the recommended protocols. DNA was quantified using the Qubit method [22].

Shotgun libraries were constructed as described in the Illumina Shotgun Sequencing Library Preparation TruSeq DNA PCR-Free HT Library Prep protocol [23]. Shotgun libraries were sequenced in Illumina Next Seq500. The shotgun library was sequenced in the Illumina NextSeq500 platform with a paired-end configuration and reads of 75 bp were generated.

### Taxonomic analysis

For WMS taxonomic analysis of the reads, we used the Kraken 2 specific database based on k-mer spectra from complete RefSeq genomes and the National Center for Biotechnology Information (NCBI) nt database (downloaded 7 September 2021) [24]. Annotation tables were formatted for use with the R ggplot2 library to generate bar stacked graphs at different taxonomic levels. The integrated matrices obtained for the four samples were written using R, bash, Perl and Python and are available at https://github.com/jenniferlu717/KrakenTools.

### Diversity analysis

Statistical analyses were performed using R.v-3.6.2. Principal coordinate analysis (PCoA) was performed using the Bray–Curtis dissimilatory index to calculate a distance matrix relative to the taxa abundance group at the genera level across all stages. Data visualization was performed using the pragma and vegan packages [25].

### Metagenomic assembly and functional analysis

Before assembly, maize sequences were removed using the BWA tool v.0.7 [26] on the reference genome of *Zea mays* (assembly B73 RefGen_v4). A *de novo* assembly with the remaining reads was performed for each fermentation time point using MEGAHIT v1.1.1–2 with the default parameters [27]. The ORFs for each time were predicted using the Prodigal-v2.6.3 tool [28]. The CAZy database-V9 was used for carbohydrate-active gene identification using HMMER-3.2.1 [29] (hmmscan cut-offs: E-value <1e-15, coverage >0.35) for annotated CAZyme domain boundaries according to the HMM database dbCAN2 [30] CAZyme domain-V9. The substrate specificities of CAZymes were inferred by manual inspection of CAZy (www.cazy.org). Metabolic pathways were predicted against the Kyoto Encyclopaedia of Genes (KEGG GENES) database using the Ghost-KOALA-v.2.2 tool [31].

### Metagenomic assembled genomes (MAGs), taxonomic classification and functional annotation

MAGs were reconstructed, refined and annotated using the Squeeze-Meta pipeline v.1.3.1 [32] (parameters: megahit, coassembly, doublepass, lowmem, miniden=60). Genomic bins with completeness <75 % and contamination >10 % were discarded. Good-quality bins were classified taxonomically with GTDB-Tk v2.1.0 against the GTDB database v-207 [33]. The bins were assigned taxonomically using GTDB-tk to try to identify strains at the species level (ANI >95 %). CAZyme modules and CAZyme gene clusters (CGCs) were annotated using the dbCAN2 metaserver [30] [hmmscan cut-offs: E-value <1e-15, coverage >0.35; DIAMOND cutoffs: E-value <1e-102, Hotpep (frequency >2.6, hits >6), CGCFinder (distance<=2, signature genes=CAZyme+ TC)].

KEGG orthologue assignment based on MAG profile HMM was predicted against the KEGG GENES database using KofamScan v1.3 [34]. Pathways for essential amino acids and vitamins were predicted using the KEGG-Decoder V1 tool [35].

The integrated matrices obtained for the four samples were written using R, bash, Perl and Python; the scripts are available at http://github.com/Ales-ibt/Metagenomic-benchmark.

## Results and discussion

### Taxonomic profile and beta diversity of pozol samples

Pozol fermentation is a complex process that mainly involves bacteria of the phyla Firmicutes, Actinobacteria and Proteobacteria [11]. A metagenomic analysis was performed at four key time points during pozol fermentation to understand the dynamics of the bacterial community throughout the course of the process.

**Fig. 2.**
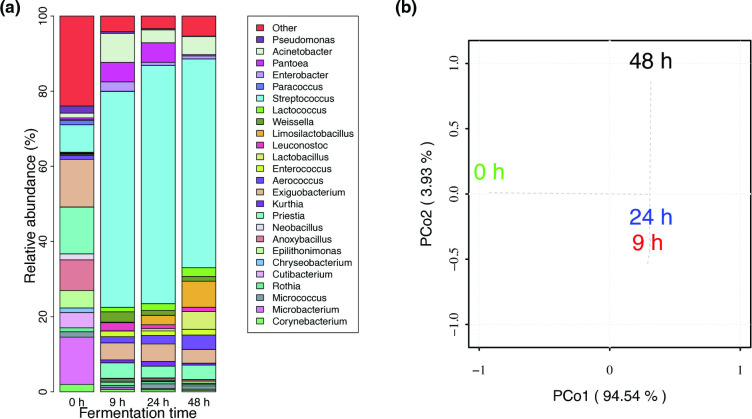
(a) Relative abundance of the genera throughout pozol fermentation according to shotgun analysis with Kraken 2 and the RefSeq and nt NCBI databases. The genera represented as ‘other’ are the sum of all genera <1 % of relative abundance at all fermentation times at the genus level. (**b)** Beta diversity at the genus level for the four fermentation times of pozol. Principal coordinate analysis (PCoA) of a Bray–Curtis dissimilarity matrix calculated at the genus level abundance annotated by Kraken 2. The first principal coordinate accounts for the largest possible amount of variation in the data and the second principal coordinate accounts for the second largest amount of variation.

The reads from each of the four fermentation stages of the sampling were analysed to define their taxonomic profiles. The most abundant phylum was Firmicutes, followed in a lower proportion by Actinobacteria and Bacteroidetes (Fig. S1, available in the online version of this article). From these phyla, the genera *Exiguobacterium *, *Priestia *, *Acinetobacter* and *Streptococcus* were present in significant abundance throughout fermentation (Fig. 2a, Table S1). Sixty per cent of all reads found in our four fermentation times belonged to *Streptococcus* (Table S2). A principal coordinate analysis (PCoA) of the relative abundance of bacteria at the genus level showed a clear separation between 0 and 48 h (91 % variance explained in CoA1) and similar abundance patterns between 9 and 24 h (Fig. 2b).

This finding is consistent with spontaneous fermentation, as the microbial community is rather heterogeneous at 0 h, and as fermentation proceeds, *Streptococcus* increases throughout the fermentation process and becomes the dominant LAB genus at the end of the process. In contrast, *Exiguobacterium* and *Bacillus* decrease as fermentation proceeds. *Anoxibacillus* is present at the beginning of fermentation and decreases as fermentation takes its course. *Leuconostoc* and *Weisella* have a relevant presence in the early stages of fermentation, particularly at 9 h. *Limosilactobacillus* starts to be significantly abundant at 24 h and continues to increase at 48 h. *Lactobacillus* begins to increase in abundance at 24 h and becomes significant at 48 h. Previous work showed that there is a shift in the evolution of the pozol microbiome after 24 h [13] marked by heterofermentative bacteria; it is suggested that the presence of an increase in acidification of the maize dough could be due to the genera *Lactobacillus* and *Enterococcus *. In general, the evolution of pozol microbial diversity described in this work and its final condition is in accordance with the historical reports of taxonomic identifications, including traditional microbiological approximations [10] and more recent proteomic approaches [36] (Fig. 1).

An interesting observation is that the microbial diversity that defines pozol is common in several cereal-based fermented foods around the world [37]. In this context, the genus *Exiguobacterium,* not previously reported in pozol, was found throughout fermentation and has only been identified in saeu-jeotgal, a Korean fermented food obtained from shrimp and used as a sauce in Korean cuisine [38]. *Exiguobacterium* may be incorporated into corn in the nixtamalization process, due to its alkalophilic nature combined with its efficient growth on starch [39, 40], while its permanence during fermentation can be explained by its efficient ß-glucosidase activity on a continuous carbon source [41].

### Comparative functional analysis of pozol samples

Pozol is known to have been a rich food in the Mayan diet, due to its nutritional properties, which include greater yield of some amino acids and vitamins than its raw mass [20].

**Fig. 3.**
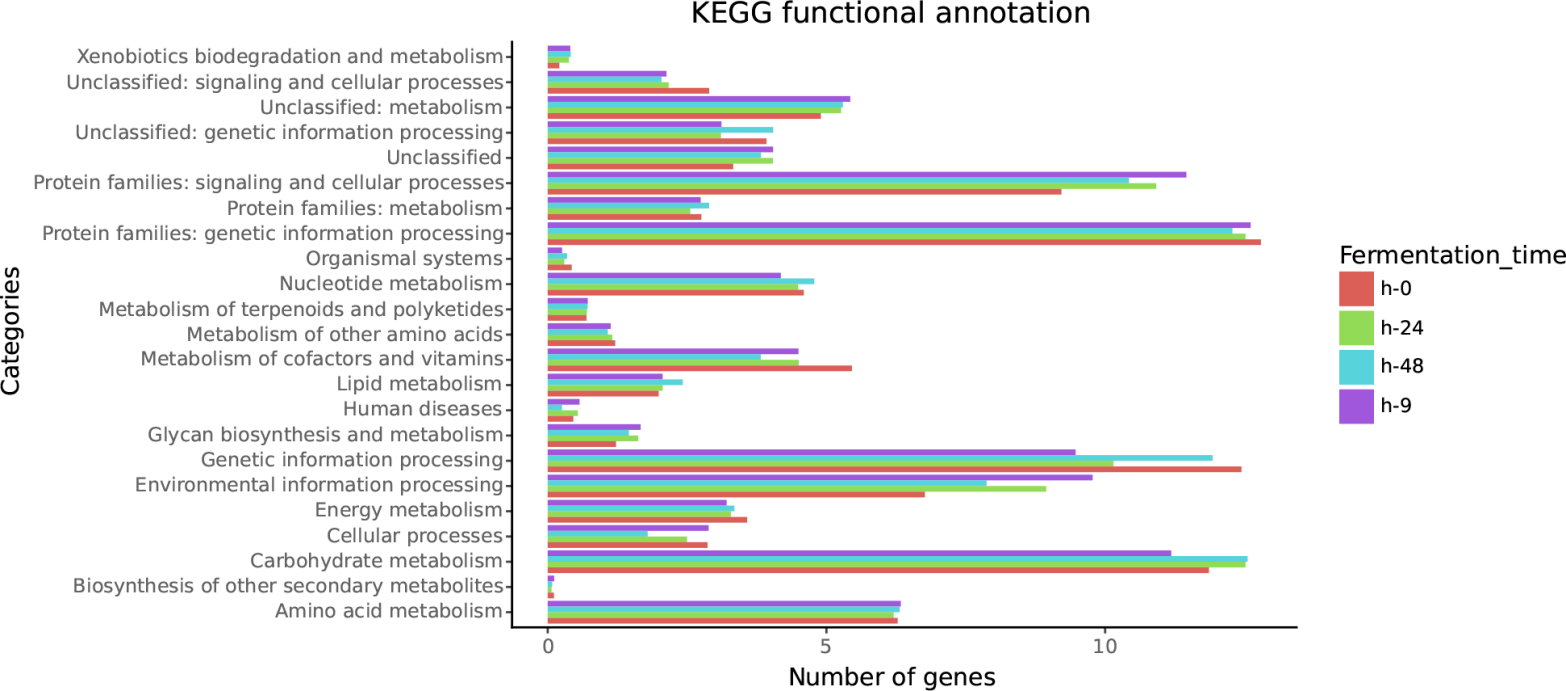
Percentage of sequences annotated for each of the KEGG categories in the four fermentation times.

To determine the metabolic potential of pozol, we annotated its metabolic capacity. After elimination of maize DNA, reads were assembled into contigs and annotated. We assigned more than 40 % of the predicted ORFs at our four fermentation time points to the distinct categories of the KEGG catalogue. The categories that showed the most genes annotated were carbohydrate metabolism, genetic information processing, signalling and cell processes at all times of fermentation (Fig. 3).

A reconstruction of the main metabolic pathways of central carbon, amino acid, lipid cofactor and vitamin metabolism was carried out. Modules of the essential amino acids threonine (M00018 of KEGG modules), lysine (M00016 and M00525 of KEGG modules), isoleucine (M00019 and M00570 of KEGG modules), arginine (M00844 and M00845 of KEGG modules), tryptophan (M00023 of KEGG modules) and riboflavin (M00125 of KEGG modules) biosynthesis pathways were annotated through pozol fermentation. Except for arginine and tryptophan, complete modules were found throughout the whole pozol fermentation process. At 0 h, KEGG modules M00023 for tryptophan and M00844 for arginine biosynthesis were each missing one block, and KEGG module M00845 for arginine was found with a block missing at all times of fermentation (refer to the Github repository for full KO annotation).

We annotated the genes for specific pathways of amino acid and vitamin biosynthesis. The presence of complete biosynthesis modules corresponding to essential amino acids such as threonine, lysine, isoleucine, tryptophan, arginine and vitamin riboflavin was found throughout the fermentation process. This result could confirm the fact that amino acids and protein content have been found to be more abundant in pozol-fermented dough than in corn after nixtamalization in the first 9 h of fermentation [8]. The biosynthesis of these molecules by LAB and their relationship with the nutritional potential of fermented foods have been the subject of various studies [18, 42].

### Comparative analysis of carbohydrate-activated enzymes (CAZymes) from pozol samples

It is known that the main source of carbon in the nixtamalized dough of pozol, which is used by amylolytic bacteria (ALAB) such as *Enterococcus *, *Lactococcus* and *Streptococcus* found in pozol, is starch, but there is a hypothesis that the use of hemicellulose, which becomes available after the nixtamalization process, could be another carbon source for microbial growth. To consider this, the CAZyme content in our metagenome samples was studied.

**Fig. 4.**
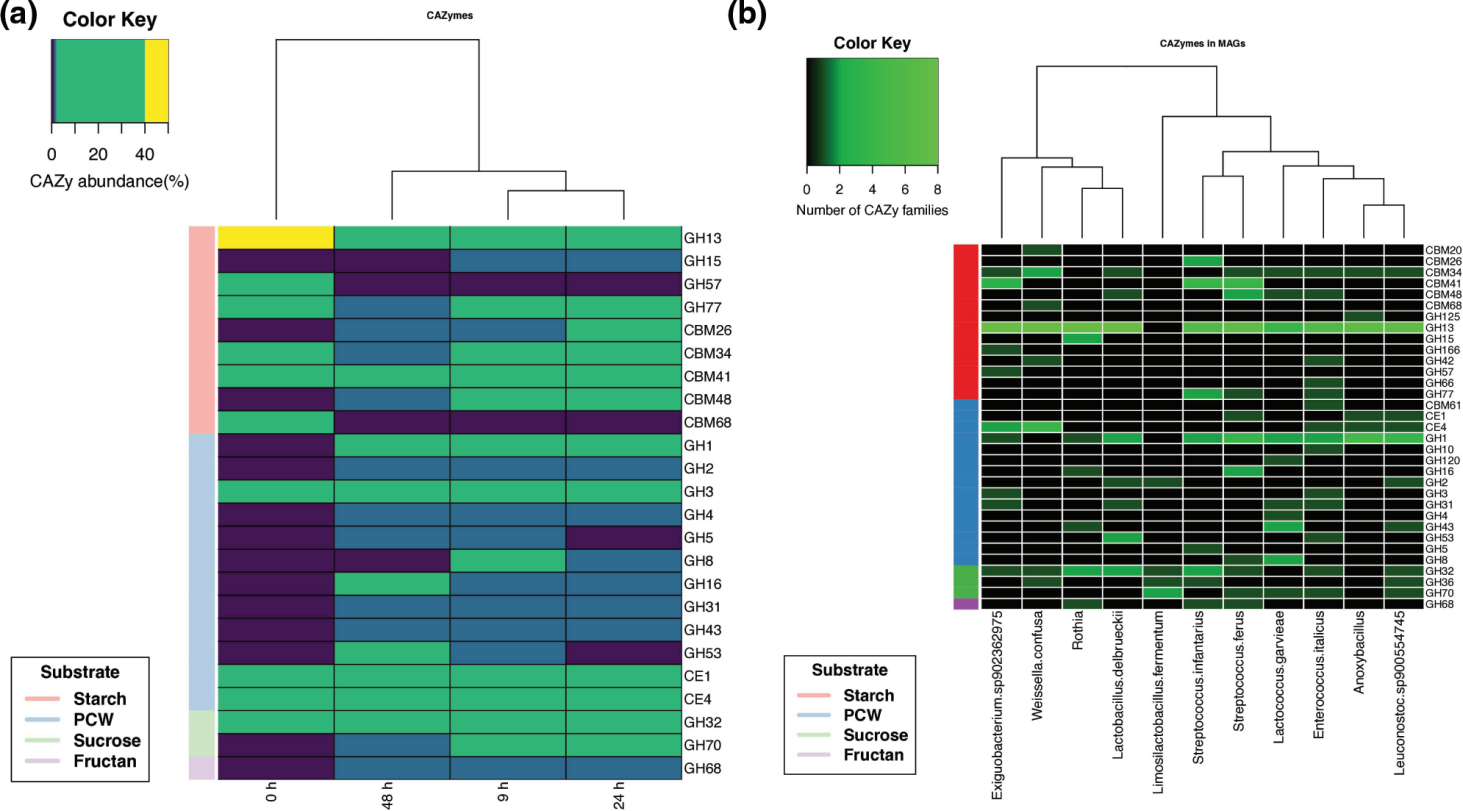
(a) Heatmap of relative abundance (%) of the CAZyme genes families targeted for the recognition and degradation of carbohydrates in pozol fermentation. Side colour labels for the main carbohydrate in that family target: starch, red; plant cell wall (PCW), blue; sucrose, green; fructose, purple.** (b)** Heatmap of the CAZyme gene family counts targeted for the recognition and degradation of carbohydrates in MAGs. Labels with colour codes for the main carbohydrate targeted by the family: starch, red; plant cell wall (PCW), blue; sucrose, green; fructose, purple. MAG names according to the GTDB database v-207 annotation.

We classified sequences within the six classes of the CAZy database involved in the synthesis, degradation and recognition of carbohydrates, glycoside transferases (GTs), glycoside hydrolases (GHs), carbohydrate esterases (CEs), carbohydrate-binding modules (CBMs), polysaccharide lyases (PLs) and auxiliary activities (AAs). These sequences were used to construct an abundance matrix throughout the fermentation process. We annotated the genes as 45 GTs, 72 GHs, 12 CEs, 17 CBMs, 13 AAs and 7 PLs families through fermentation. We discarded all modules that did not target the degradation of carbohydrates present in corn dough to examine specifically the genes encoding CAZymes involved in the degradation of carbohydrates found in pozol, leaving us with families involved in starch, maize plant cell wall (PCW), fructan and sucrose degradation (Fig. 4a) (refer to the Github repository for full gene counts encoding CAZymes).

We found that the most abundant GH families targeting starch are GH13 and GH77, members of the α-amylase clan GH-H, which includes enzymes that act on substrates containing α-glucoside linkages. The carbohydrate-binding modules CBM26, CBM34, CBM41, CBM48 and CBM68 were found abundantly; the CBMs form families that bind starch among other carbohydrates (http://www.cazy.org; https://www.cazypedia.org/) (Fig. 4a).

The gene families GH, GH3, GH8, GH16 and GH53 were abundant in pozol fermentation; these families are known to contain enzymes involved in the hydrolysis of components of the PCW, where (1→4)-β-d-glucans are predominant. GH3 is known to perform a variety of functions, including cellulosic biomass degradation and plant cell wall remodelling. GH8 is known to cleave β−1,4 linkages of β−1,4 xylans or xylooligosaccharides and therefore includes cellulases and xylanases. GH16 enzymes are active in plant polysaccharides and include endoxyloglucanases. (http://www.cazy.org; https://www.cazypedia.org/). Carbohydrate esterases CE1 and CE4, known to contain enzymes involved in the deacetylation of xylans and xylo-oligosaccharides that could potentially act on the PCW, were abundant in the pozol metagenome (http://www.cazy.org; https://www.cazypedia.org/) (Fig. 4a).

Modules of GH32 and GH70, which are the most abundant families that include enzymes responsible for sucrose hydrolysis, are prevalent during pozol fermentation. GH32 contains activities such as invertase (EC 3.2.1.26). It also contains enzymes that hydrolyse fructose-containing polysaccharides such as inulinases and levanases. The activities of members of the GH70 family also involve sucrose hydrolysis, but they are classified primarily as glucosyltransferases, as they are also capable of synthesizing exopolysaccharides. This family includes dextransucrase and alternansucrase, among other transferases common to many fermentation products (http://www.cazy.org; https://www.cazypedia.org/). It also includes enzymes such as levansucrases or inulosucrases that catalyse levan or inulin-type fructans, as well as fructooligosacharides (FOS), and can use fructan as a donor substrate (http://www.cazy.org; https://www.cazypedia.org/).

The CAZyme genes were found at any fermentation time, always constituting more than 18 % of all the annotated genes, mainly associated with the degradation capacity of carbohydrates, such as starch, plant cell wall polysaccharides, sucrose and fructan, by bacteria found in pozol. Unsurprisingly, CAZy families involved in starch and maltodextrin degradation are present throughout the fermentation process, since starch, the main corn carbohydrate, constitutes almost 70 % of the total pozol dough [11]. Consequently, the core microbiota responsible for corn fermentation is expected to have this metabolic capacity, as has already been shown in *S. infantarius *, isolated from pozol [14, 43]. On the other hand, while the alkaline thermal treatment associated with nixtamal removes most soluble sugars, it also predigests complex insoluble carbohydrates from the plant cell wall, increasing the availability of arabinoxylan, the main component of the hemicellulose present in fermented maize dough [44, 45]. This is consistent with the finding of abundant CAZyme gene families that act on PCW throughout fermentation. The enzymes of these families have metabolic potential to release d-xylose residues from arabinoxylan, as well as to deacetylate and remove arabinose from xylans and xylooligosaccharides [46, 47]. In this context, it is remarkable to find a high abundance of members of the GH3 family at all fermentation times.

Overall, these results allow us to infer constant ß-xylosidase and arabinofuranosidase activities, like those already identified in strains of *S. infantarius* [16] and *W. confusa* [21]. Furthermore, the prevalence of the CAZy families GH1, GH8, GH5 and GH6, involved in the degradation of cellulose and cellulose derivatives, is an indication that this maize substrate could also be available for degradation by the pozol community through nixtamalization. Until now, cellulose has not been considered to be an important carbohydrate source in pozol fermentation, so future analyses involving evaluation of the cellulolytic activity of pozol-isolated strains are necessary.

Genes encoding well-characterized enzymes acting on sucrose, such as those in the GH70 family, common in LAB of the genera *Streptococcus *, *Leuconostoc *, *Weisella* or *Lactobacillus* [48], were also found through fermentation. Hence, although sucrose is not a substrate present in significant concentrations, it could play a role in the metabolism of the microbiome present in pozol feeding species without amylolytic or cellulolytic machinery.

It should be noted that the GH70 family houses enzymes known to synthesize exopolysaccharides [49]. These sugars include dextrans and other glucans that have benefits related to their rheological properties that favour the taste and texture of fermented food [50]. This functional inference can be supported by the detection of exopolysaccharides in pozol [51] and although we know that various organisms of the microbial community have these capabilities, they have been directly demonstrated in *W. confusa* strains and *Leuconostoc citreum* isolated from pozol [21, 52]. Therefore, an interesting novelty is the detection, in addition to members of the GH70 family, of enzymes of the GH68 family and CBM66, an indirect indication of the presence of fructans, the strongest soluble fibre and prebiotic sugars that favour the beneficial LAB of the human gut [53, 54].

### Taxonomic classification and functional annotation of assembled MAGs

A total of 11 bins were reconstructed using the Squeeze-Meta pipeline. Based on contamination parameters with the CheckM [55] and DasTool [56] software tools, four high-quality bins (>90 % complete, <10 % contamination) and seven good-quality bins (>75 % complete, <10 % contamination) were reconstructed and annotated taxonomically. Two MAGs were assigned at the genus level and nine at the species level (Table S3). We can conclude that these bins correspond to the most abundant genera found in pozol (Fig. 2, Table S3) [21].

These MAGs were annotated taxonomically as *Exiguobacterium *, *Leuconostoc *, *Enterococcus* (*E. italicus)*, *Streptococcus* (* S. ferus *, *S. infantarius *), *Lactobacillus* (* L. delbrueckii *), *Limosilactobacillus (L. fermentum*), *Anoxybacillus* spp., *Weissella (W. confusa)* and *Lactococcus* (* L. garvieae *). All annotated species belong to genera with a relevant presence in pozol fermentation. It should be noted that *Lactococcus* spp., *Exiguobacterium* spp., *Enterococcus* spp., *Leuconostoc* spp., *S. infantarius *, *L. fermentum *, *L. delbrueckii* and *W. confusa* were previously reported in pozol fermentations. [12–16, 52].

MAGs correspond to species common to fermented foods all over the world and not only based on corn. *Exiguobacterium* spp. are interesting species to observe, as they are found throughout fermentation. *Exiguobacterium* strains have been isolated in food processing plants and various other environments where important temperature changes can occur (-12–55 °C) and can grow within a wide range of pH values (5–11) [40], probably explaining their presence in pozol.

Another interesting strain is *L. delbrueckii,* a bacterial species used extensively in the food industry, which has been reported in traditional African fermented maize poto-poto [57], while *W. confusa* is also the main bacterium found in the fermented alcoholic beverage chicha from the northeast of Argentina [58]; both species have already reported in other maize fermentations. In the same context, *Lactobacillus* spp. have been found in more refined fermented products, such as the three most important cheeses produced in Italy, Grana Padano, Parmigiano Reggiano and Mozzarella [59]. *L. garvieae* has been isolated from vegetables, meat, raw milk and natural fermentation of different artisanal cheeses [60].

*S. infantarius* has been isolated frequently from pozol and is one of the most important micro-organisms in pozol fermentation [16], but also in other spontaneously fermented beverages such as Kenyan camel milk (suusac) [61], as well as in traditionally fermented dairy and plant products in Europe. It appears to also play a central role in spontaneous dairy fermentations in Africa.

Another *Streptococcus* species, *S. ferus,* has been isolated from the oral cavity of wild rats found in sugar cane plantations [62]. Finally, *Anoxibacillus* spp. have been found in high temperature habitats and processed foods such as gelatine, and according to previous studies, it can grow over a wide range of temperatures and pH values (30–72 °C and from 5.5 to 10.0, respectively) [63].

### Carbohydrate profile of MAGs found in pozol

The functional annotation of CAZymes was performed using dbCAN2 [30]. CAZyme families involved in the degradation of pozol substrates were annotated and counted in each bin. Again, we discarded all modules that did not target the degradation of carbohydrates present in pozol dough, retaining only members of the CAZyme family involved in starch, maize PCW, fructan and sucrose degradation (Fig. 4b) (refer to the Github repository for full gene counts encoding CAZymes). Based on these annotations, we found at least one module that was intended to degrade starch, PCW, sucrose, or fructan in all bins. The bins corresponding to *W. confusa *, *Exiguobacterium* sp., *S. ferus *, *S. infantarius* and the genus *Rothia *, respectively, contained CAZyme families targeting all substrates. Families degrading starch and PCW were found in all bins except in *L. fermentum *. Sucrose or fructan modules were found in all bins except *L. garvieae. L. fermentum* only contained CAZyme families associated with sucrose and fructan carbohydrates (Fig. 4b).

**Fig. 5.**
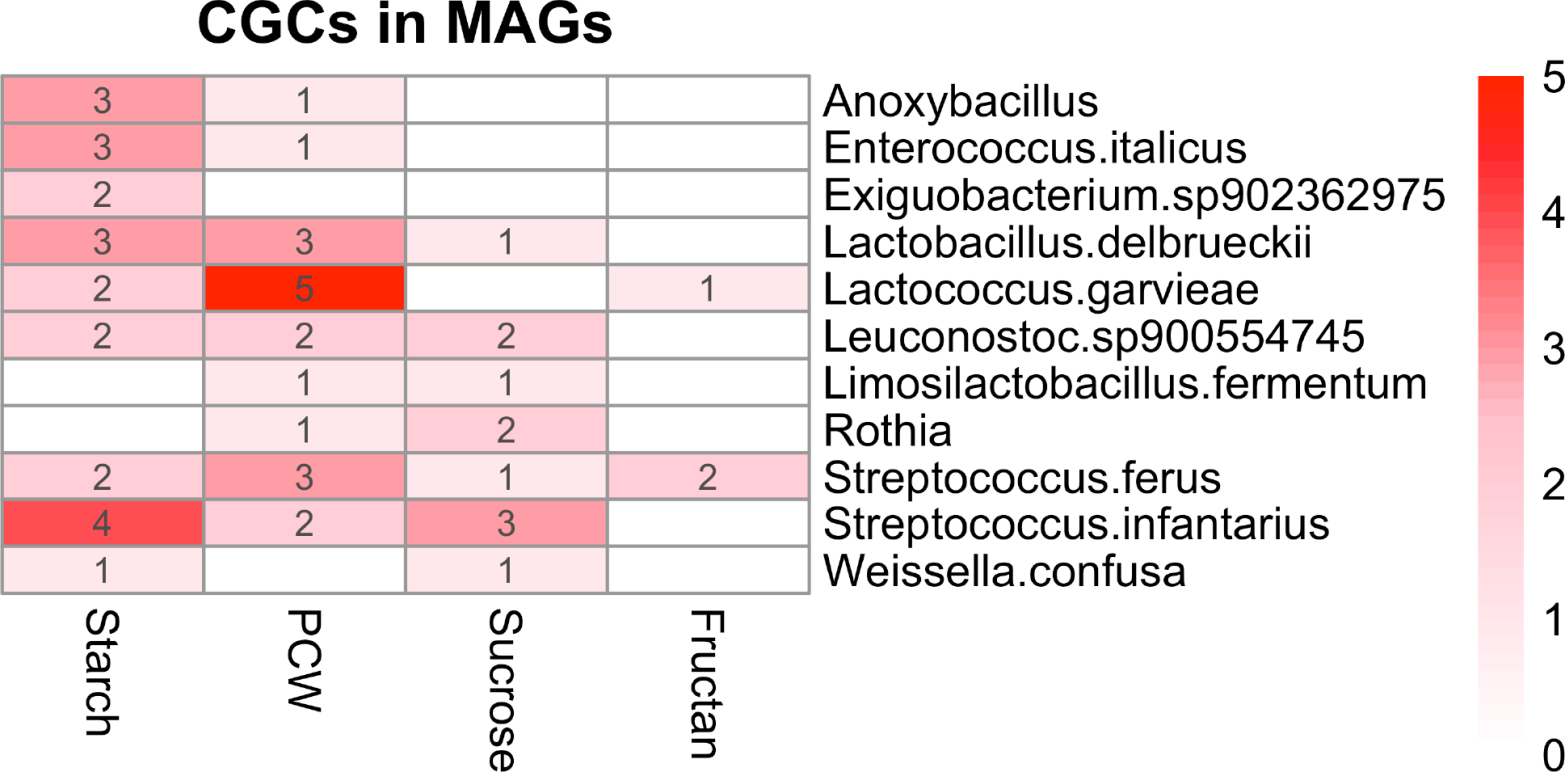
Heatmap of CAZyme gene clusters (CGCs) counts targeted for the recognition and degradation of the main carbohydrates found in MAGs. The heatmap indicates the number of CGCs found in each MAG targeting a specific carbohydrate showing its relative distribution. MAG names according to the GTDB database v-207 annotation.

CGCs were found in all bins. All, except *L. fermentum,* had CGCs involving CAZymes families targeting starch degradation; *Exiguobacterium* sp. and *Anoxybacillus* did not have CGCs involving CAZymes that target PCW. CGCs involving sucrose and fructan degradation were not found in *E. italicus* and *L. garvieae *. The bins that had more CGC targeting pozol carbohydrates were *S. ferus, S. infantarius* and *W. confusa* with eight, nine and eight, respectively (Fig. 5).

We highlight the metabolic potential of the reconstructed MAGs to degrade the four main carbohydrates present in nixtamalized corn dough. In fact, we found carbohydrases and carbohydrate-binding modules involved in the utilization of starch, hemicellulose, cellulose and sucrose. Pozol is a fermented acid beverage made from nixtamal, which separates this fermentation from others because of the low concentration of mono- and disaccharides of corn reduced during the alkali thermal process of nixtamalization. For this reason, starch is the main carbohydrate available for lactic acid fermentation. All bins, except those assigned taxonomically to *L. fermentum,* showed metabolic potential to recognize and metabolize this carbon source. However, *L. fermentum* has genes encoding enzymes involved in sucrose metabolism that are present in fermented maize dough. *Lactobacilli* were found to be more abundant after 24 h and their sudden growth could be a consequence of the presence of oligosaccharides and disaccharides previously broken down by other species that had a relevant presence throughout fermentation. *L. delbrueckii* has the metabolic potential to degrade the four carbohydrates present in the pozol dough. CGCs involving starch degradation, hemicellulose plant cell wall and sucrose were found (Fig. 5). The presence of these two genera could explain the acidification of the beverage and the community change.

The genus *Exiguobacterium* was detected at all times of fermentation. It has modules aimed mainly at all possible carbohydrates found in pozol and CGCs aimed at starch (Fig. 5). *Exiguobacterium* sp. is one of the pozol colonizers and a species that shapes the community, as it has the metabolic potential to degrade all carbohydrates found in pozol.

*Anoxybacillus* sp. was abundant at the beginning of the fermentation process and then decreased as fermentation went on. This suggests that *Anoxibacillus* sp. is also is a colonizer bacterium that survives the nixtamalization process due to its tolerance to a wide range of temperatures and pH, but it cannot compete with the bacterial consortium. The annotation results showed that it has genes for starch degradation (Fig. 4).

*Enteroccocus* is a generalist that increases through fermentation. *Enterococcus* spp. are homofermentative bacteria, and species such as *E. italicus* have been found in Italian cheeses and other fermented foods [64].

*Weisella* and *Leuconostoc* spp. were found to be more abundant after 9 h of fermentation. *W. confusa* has genes that encode the utilization of three carbohydrates present in fermentation and has the highest number of CGCs found that target plant cell wall hemicellulose, while *Leuconostoc* has genes that encode enzymes involved in the hydrolysis and deacetylation of xylans and xylo-oligosaccharides. *Lactococcus* was found to be more abundant at 24 h. *L. garviae* has genes and CGCs for the metabolism of starch hydrolysis and hemicellulose hydrolysis and the deacetylation of xylans and xylo-oligosaccharides. Therefore, these species must be involved in plant cell degradation (Fig. 5).

*Streptococcus* was the most abundant genus throughout the process. The two reconstructed species (* S. infantarius* and *S. ferus *) had CAZymes for the binding and degradation of starch, hemicellulose fructan and sucrose hydrolases, and CGCs for the degradation of the main carbohydrates. These CGCs must give *Streptococcus* an ecological advantage, explaining its significant advantage over other genera and the ability to survive fermentation (Fig. 5). Furthermore, complete pathways for the biosynthesis of amino acids were found were found in a higher proportion in pozol than in its raw mass in the MAGs we reconstructed (Fig. S2). *S. infantarius* and *S. ferus* possess complete pathways for the biosynthesis of most of the essential amino acids and vitamins that have been highlighted in pozol.

## Conclusions

The core microbiota for pozol fermentation was confirmed by metagenomic analysis. The bacteria of the phylum Firmicutes, more specifically lactic acid bacteria (LAB), carry out this complex traditional fermentation using several carbon sources as substrate. This study complements a previous diversity approach using the 16S ribosomal gene, where 18 genera had been identified as the most dominant [21]. The samples used in this study were divided into three batches for parallel studies, one for metaproteomic analyses [9], one for 16S metagenomic analyses [21] and one for shotgun metagenomics (this study). There was significant overlap in the predominant genera found with the three distinct techniques. Furthermore, the population dynamics showed strong similarities in all instances [21, 9]. The same 18 most abundant genera were also identified with the 16S metagenomic approach [21].

Despite regional variations in pozol from Chiapas and Tabasco, *Streptococcus* and the other abundant genera identified here have been consistently identified in different studies [8, 9, 11, 13, 14, 65].

It was previously established that these bacteria were able to inoculate the dough and were preserved by fermentation. *Streptococcus* was found to be the predominant genus in fermentation. We have been able to assemble genomes of the most abundant genera through fermentation with the metabolic potential to consume starch, hemicellulose, fructans and sucrose as the main possible substrate targets to sustain fermentation, indicating which substrates sustain pozol fermentation. Furthermore, this metabolic potential also extends to cellulose, which was not considered a carbon source in pozol fermentation.

Our findings show that the bacteria present in fermentation utilize various carbon sources that provide nutrients for the whole community, degrading polysaccharides such as starch, hemicellulose, xylan, arabinoxylan, fructans, sucrose and even cellulose into dimers and monomers readily available for the consortium. From our findings, *Streptococcus* plays the most important role; consequently, we were able to metagenomically assemble two species from this genus. This genus has metabolic potential for the degradation of the various available carbon sources: starch, hemicellulose arabinoxylan fructan and sucrose. Other genera, such as *Exiguobacterium* and *Anoxybacillus,* participate as starter cultures, while *Lactobacillus* spp. *and Limosilactobaillus* spp. belong to shifting communities that will later acidify the dough. Bacteria of the genera *Weisella* and *Leuconostoc* are present in this fermentation because they can metabolize partially degraded hemicellulose available after nixtamalization. Finally, bacteria of the genus *Enteroccocus* increase as fermentation proceeds.

The same can be said about the soluble fibre provided by the dextrans and fructans present, including their prebiotic role. The presence of genes corresponding to various amino acid biosynthesis modules, as well as riboflavin, is found in a higher proportion in fermented than in raw maize dough, confirming the important nutritional properties of this traditional beverage. Additionally, biosynthesis of these essential amino acids and vitamins occurs in most species reconstructed in this work. If nixtamalization is a chemical process that improves the nutritional value of corn, fermentation takes this staple food one step further in terms of quality and nutraceutical value. The study of spontaneous fermentations with ‘omic’ tools provides new interpretations and a deeper understanding of the process and, as such, reveals new avenues of research.

Regional and longitudinal studies should allow us to understand the general patterns of the community structure, the dynamics of spontaneous fermentation and the effects of regional maize varieties, nixtamalization and processing.

## Supplementary Data

Supplementary material 1

Supplementary material 2

Supplementary material 3
