# Supplementary figures and images for: Analysing the dynamics of the bacterial community in pozol, a Mexican fermented corn dough
Source: Microbiology (Reading). 2023 Jul 6;169(7):001355. doi: 10.1099/mic.0.001355 (PMC10433422; doi:10.1099/mic.0.001355)

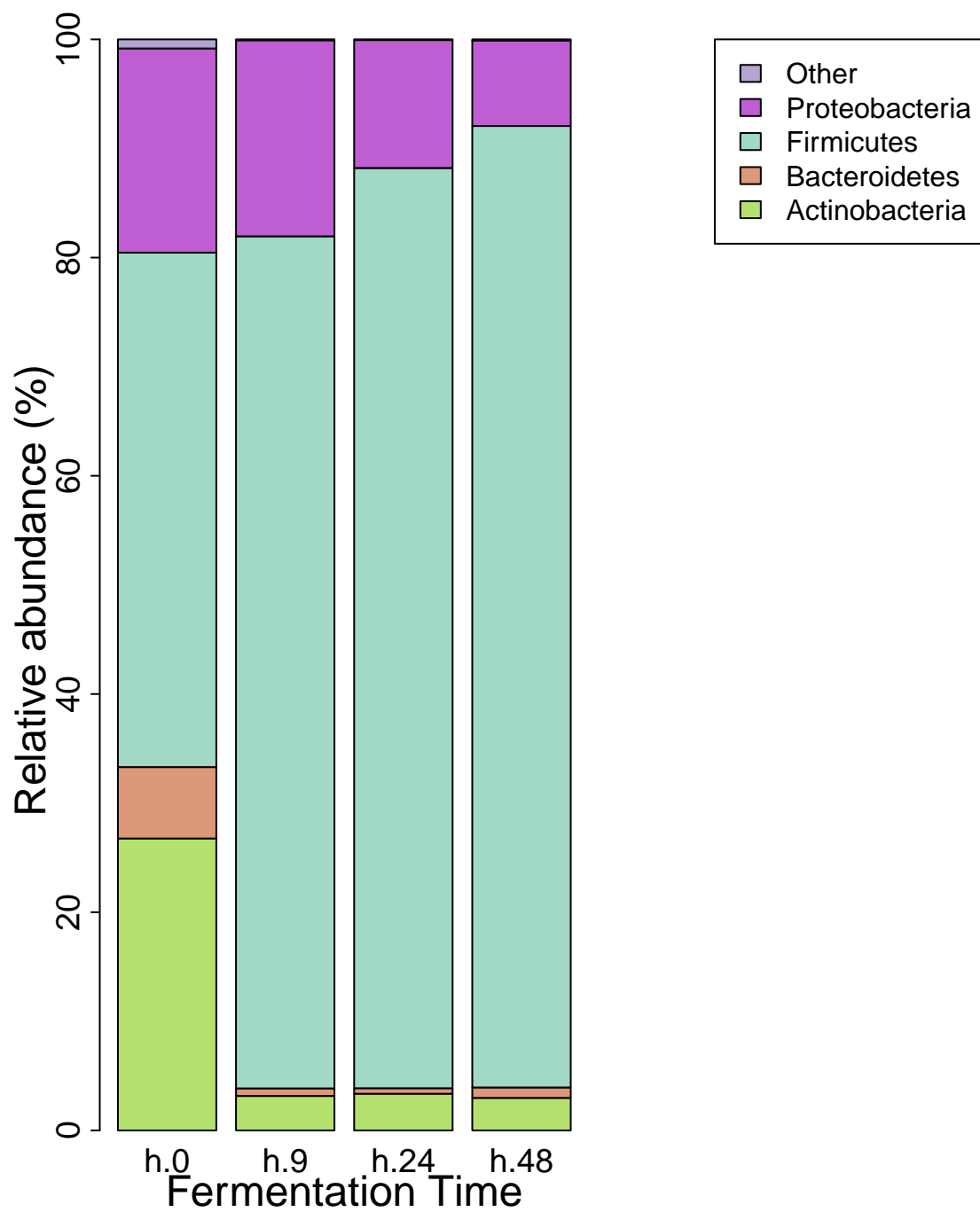

Supplement: Supplementary material 1 [file mic-169-1355-s001.pdf]

Completeness of Biosynthesis pathways of amino acids and vitamins in MAGs found in pozol

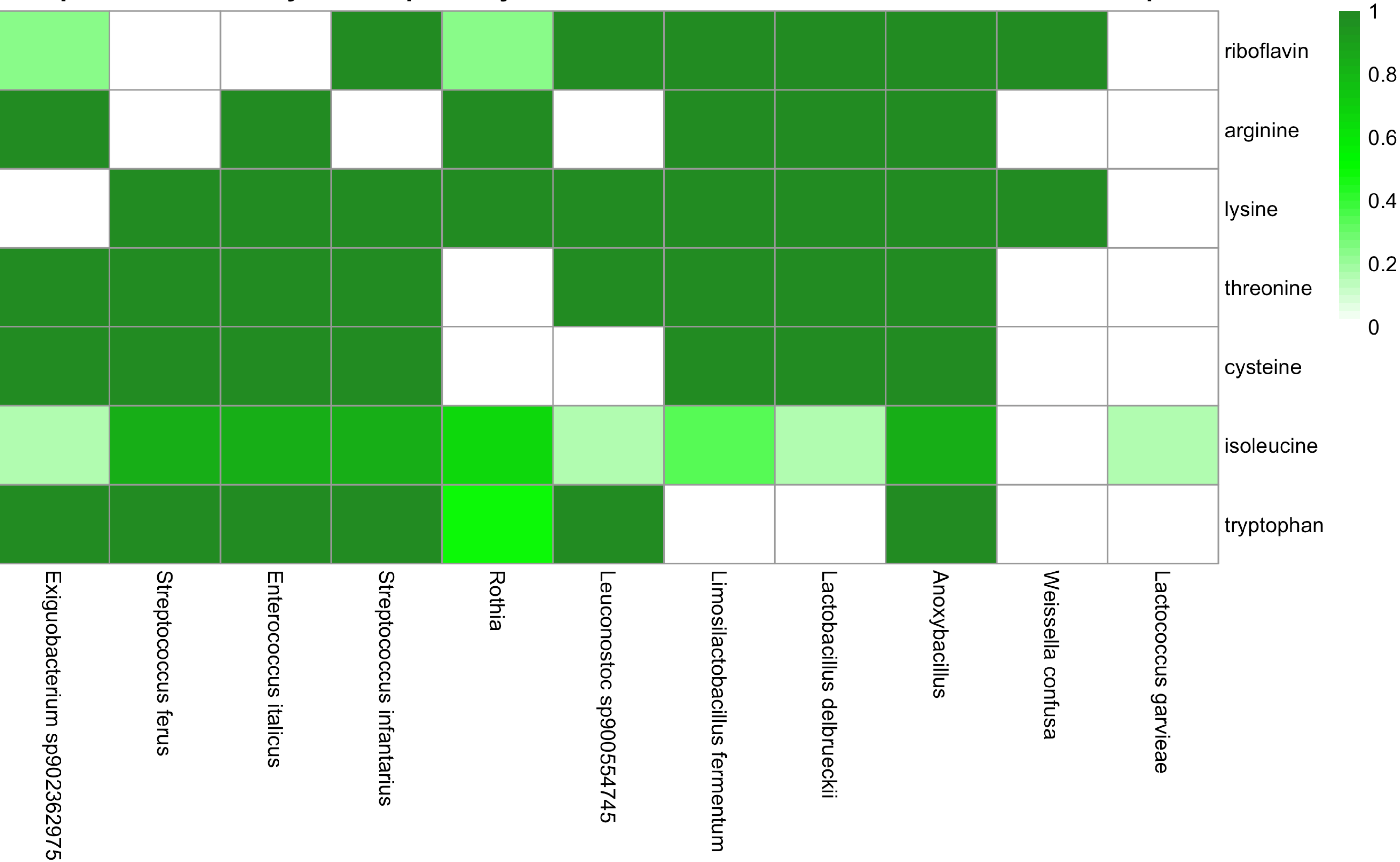

Supplement: Supplementary material 2 [file mic-169-1355-s002.pdf]
