# Supplementary material for: Analysing the dynamics of the bacterial community in pozol, a Mexican fermented corn dough
Source: Microbiology (Reading). 2023 Jul 6;169(7):001355. doi: 10.1099/mic.0.001355 (PMC10433422; doi:10.1099/mic.0.001355)
Supplement: Supplementary material 3 [file mic-169-1355-s003.pdf]

Analyzing the dynamics of the bacterial community in "pozol", a Mexican fermented corn dough.

López-Sánchez Rafael[1], Hernández-Oaxaca Diana[1], Escobar-Zepeda Alejandra[2], Ramos Cerrillo Blanca[1], López-Munguía Agustín[1] and Segovia Lorenzo[1].

Supplementary Table 1. Most abundant taxa genera (%) across the four times of pozol fermentation.

Taxonomy	h-0(%)	h-9(%)	h-24(%)	h-48(%)
Corynebacterium	1.957185	0.625773	0.545569	0.407896
Microbacterium	12.586191	0.452678	0.27637	0.303677
Micrococcus	1.432078	0.629768	1.269778	0.843859
Rothia	1.057764	0.833817	0.611517	0.463053
Cutibacterium	4.057246	0.02864	0.032981	0.027871
Chryseobacterium	1.221077	0.128859	0.123084	0.247328
Epilithonimonas	4.633907	0.408151	0.269161	0.547144
Anoxybacillus	8.170465	0.402953	0.471514	0.354952
Neobacillus	1.590972	0.085562	0.095608	0.075502
Priestia	12.429137	4.113461	3.113308	3.829683
Kurthia	0.052106	0.77417	1.266558	0.510033
Exiguobacterium	12.697399	4.536428	4.652183	3.659614
Aerococcus	1.054818	1.647686	2.268426	3.83619
Enterococcus	0.257398	1.508496	1.214007	1.497521
Lactobacillus	0.034246	0.055705	0.603866	4.753772
Leuconostoc	0.186697	2.158027	1.034684	1.117539
Limosilactobacillus	0.102738	0.135711	2.485868	6.95662
Weissella	0.069413	2.762092	1.313188	1.271623
Lactococcus	0.139194	1.170561	1.788591	2.31583
Streptococcus	7.342113	57.480544	63.420048	55.55449
Paracoccus	1.123494	0.048601	0.020177	0.017894
Enterobacter	0.260713	2.493277	0.825374	0.797964
Pantoea	0.444832	5.192732	5.19202	0.35801
Acinetobacter	1.269685	7.718908	3.413088	4.810274
Pseudomonas	1.896978	0.466291	0.334718	0.106214
**Other**	23.932154	4.141109	3.358314	5.335447

Supplementary Table 2. Most abundant taxa genera (%) of pooled reads in pozol fermentation.

Taxonomy	Percentage (%)
Streptococcus	58.0101916015469
Acinetobacter	5.24643419716044
Exiguobacterium	4.59975734835932
Pantoea	3.98714265213019
Priestia	3.88504343384883
Limosilactobacillus	2.56109292788192
Aerococcus	2.35937596856976
Weissella	1.80200806761211
Lactococcus	1.6365536332777
Leuconostoc	1.44244589131585
Enterobacter	1.41546951948665
Enterococcus	1.36007247852226
Lactobacillus	1.31534772838933
Micrococcus	0.944391112608614
Kurthia	0.885140996616515
Rothia	0.671535241023898
Microbacterium	0.671103890486205
Anoxybacillus	0.623010728851065
Corynebacterium	0.58134905219928

Supplementary Table 3. Stats of bins and their taxonomic analysis (*ANI>95%*). Bins with * were not refined with the SqueezeMeta script for removing duplicate markers.

**Genome/bin designation**	Size (bp)	No. of contigs	Proposed taxonomic Placement[a]	% completeness	% contamination	% strain heterogeneity
**maxbin.001**	2,245,440	871	*Exiguobacterium sp902362975*	76.99	0.53	73.33
**maxbin.007**	1,558,464	182	*Streptococcus ferus*	84.16	0.69	75.00
**maxbin.012**	2,489,240	356	*Enterococcus italicus*	94.95	0.00	0.00
**maxbin.013**	1,429,445	213	*Streptococcus infantarius*	84.75	0.32	33.33
**maxbin.018**	3,165,207	1,127	*Rothia*	88.01	4.47	3.57
**maxbin.020**	1,541,171	161	*Leuconostoc sp900554745*	95.02	0.2	75.00
**maxbin.024***	1,835,300	168	*Limosilactobacillus fermentum*	94.44	0.08	0.00
**maxbin.030***	1,844,352	129	*Lactobacillus delbrueckii*	95.32	0.16	0.00
**metabat2.6**	1,706,939	121	*Weissella confusa*	87.97	0.88	0.00
**metabat2.7***	1,589,068	145	*Lactococcus garvieae*	87.19	0.43	0.00
**metabat2.22**	2,135,578	115	*Anoxybacillus*	85.45	0.98	0.00
